# Supplementary material for: Culture Supernatant of Enterococcus faecalis Promotes the Hyphal Morphogenesis and Biofilm Formation of Candida albicans
Source: Pathogens. 2022 Oct 12;11(10):1177. doi: 10.3390/pathogens11101177 (PMC9608753; doi:10.3390/pathogens11101177)
Supplement: Supplementary file 1 [file pathogens-11-01177-s001.zip › pathogens-1924228-supplementary.pdf]

Supplementary files

Culture supernatant of *Enterococcus faecalis* promotes the hyphal morphogenesis and biofilm formation of *Candida albicans*

Qingsong Jiang[1,2], Qi Jing[1], Biao Ren[1], Lei Cheng[1,3], Xuedong Zhou[1,3], Wenli Lai[1,2], Jinzhi He[1,3]* and Mingyun Li[1]*

1 State Key Laboratory of Oral Diseases, National Clinical Research Center for Oral Diseases, West China School of Stomatology, Sichuan University, Chengdu, Sichuan 610041, China

2 Department of Orthodontics, West China School of Stomatology, Sichuan University, Chengdu 610041, China

3 Department of Cariology and Endodontics, West China School of Stomatology, Sichuan University, Chengdu, 610041, China

Corresponding author:
Mingyun Li, Ph.D.
Associated Professor
State Key Laboratory of Oral Diseases
West China School of Stomatology
Sichuan University
No. 14, Section 3, Renmin South Road
Chengdu, China 610041
Email: limingyun@scu.edu.cn

Jinzhi He, Ph.D.
Associated Professor
State Key Laboratory of Oral Diseases
National Clinical Research Center for Oral Diseases
Department of Cariology and Endodontics
West China Hospital of Stomatology
Sichuan University
No. 14, Section 3, Renmin South Road
Chengdu, China 610041
Email: hejinzhi@scu.edu.cn

1. Materials and methods

1.1.Co-culture of *E. faecalis* and *C. albicans*

E. faecalis stains at 1×10^7 CFU/ml were incubated for 24h. Then the bacterial cells were collected by centrifugation at 5,000 rpm for 10 min and re-suspended in fresh BHI

medium. Subsequently, *E. faecalis* and *C. albicans* were inoculated to a 24-well plate containing 0.5ml BHIS (BHI with 1% sucrose, w/v) and 0.5ml YNBB in each well. In the control group, equal volume of fresh BHI and *C. albicans* were inoculated to the plates. Both the final concentrations of *E. faecalis* and *C. albicans* were 1×10^6 CFU/ml[44]. The plates were incubated at 37°C for 24h to form biofilms. Biofilm biomass was evaluated with crystal violet staining.

1.2.Collection of *E. faecalis* intracellular contents and *C. albicans* biofilm treatment

E. faecalis stains at 1×10^7 CFU/ml were incubated for 24h. The bacteria were pelleted by centrifugation at 5,000 rpm for 10 min. Then the cell pellets were washed with sterile PBS to and re-suspended to the initial volume. The intracellular contents were extracted according to the protocol described by a previous study[45] with some modifications. In brief, bacterial cells of 5ml suspension were collected by centrifugation at 5,000 rpm for 10 min, followed by incubation with 1ml of lysozyme (25mg/ml) at 37°C for 60min. Subsequently, the cells were disrupted by sonication with a procedure of 5s pulses with 5s pause for 20min. Then the debris was removed by centrifugation at 6000 ***g*** for 10min and the supernatant was collected as the intracellular contents of *E. faecalis.*

For biofilm formation, the initial *C. albicans* suspension was diluted to 1×10^7 CFU/ml. Then, 200μl of intracellular content (equal volume of lysozyme solution for control), 700μl of YNBB and 100μl of fungal suspension were inoculated to 24-well plates. The plates were incubated at 37°C for 24h. Biofilm biomass was evaluated with crystal violet staining.

1.3. Treatment of *C. albicans* biofilm with the culture supernatant of *Streptococcus gordonii* (CSSg) and *Streptococcus salivarius* (CSSs)

S. salivarius ATCC13419 and *S. gordonii* ATCC10558 were grown in BHI broth in an anaerobic system (5% H_2, 5% CO_2, and 90% N_2) at 37°C for 24 h. Then, the bacterial suspension was adjusted to 1×10^7 CFU/ml and incubated for another 24h, followed by

centrifugation at 5,000 rpm for 10 min. Then supernatant was pipetted into another centrifuge tube and filter-sterilized using a 0.22-μm pore size filter. The pH values of CSSg and CSSs along or supplemented with equal volume of YNBB were measured. BHI was set as control. Each sample was analyzed in triplicate.

To form biofilms, the initial *C. albicans* suspension was adjusted to 1×10^7 CFU/ml in YNBB. Subsequently, 0.1 ml of the diluted suspensions of *C. albican* was inoculated to 24-well plates containing 0.5 ml CSSs + 0.4ml YNBB, or 0.5 ml CSSg + 0.4ml YNBB in each well. The negative control contained 0.5 ml BHI and 0.4 ml YNBB in each well. The plates were incubated at 37℃ for 24h in an anaerobic condition. Biofilm biomass was evaluated with crystal violet staining.

2. Results

2.1. Effect of *E. faecalis* cells on *C. albicans* biofilm formation

As shown in Fig. S1, there was no significant difference between the *E. faecalis* cells treated group and the control group. The data suggested that *E. faecalis* cells did not promote the biofilm formation of *C. albicans.*

2.2. Effect of *E. faecalis* intracellular contents on *C. albicans* biofilm formation

As shown in Fig. S2, no difference was observed after *E. faecalis* intracellular content exposure. The intracellular content of *E. faecalis* exhibited no promotion effect on *C. albicans* biofilm formation.

2.3.The pH values of CSSg and CSSs

As shown in Fig. S3, the pH values of CSSg and CSSs were significantly lower compared with BHI. However, when mixed with YNBB, no significant difference of pH was observed among CSSg, CSSs and control.

2.4. Effect of CSSg and CSSs on *C. albicans* biofilm formation

Our results showed that CSSg and CSSs exhibited no effect on *C. albicans* biofilm

formation (Fig. S4)

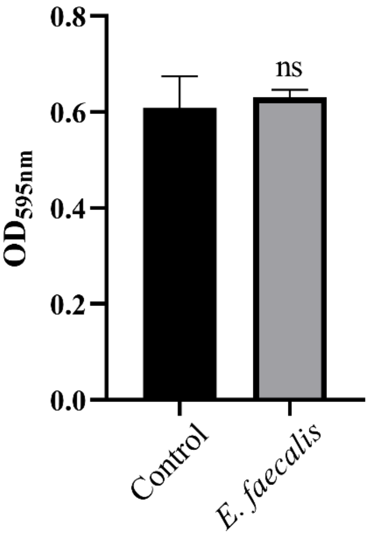

Figure S1. The effect of *E. faecalis* cells on *C. albicans* biofilm formation. The biofilm biomass was investigated by crystal violet staining. ns: no significance.

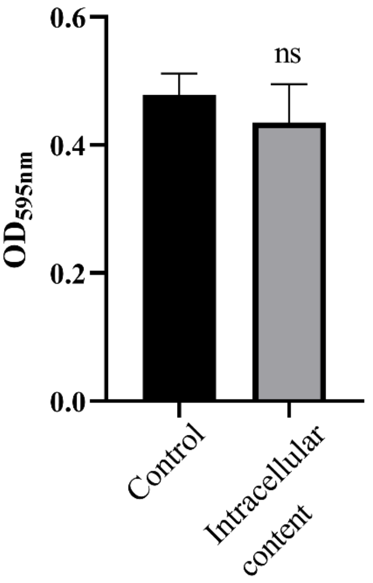

Figure S2. The effect of *E. faecalis* intracellular contents on *C. albicans* biofilm formation. The biofilm biomass was investigated by crystal violet staining. ns: no significance.

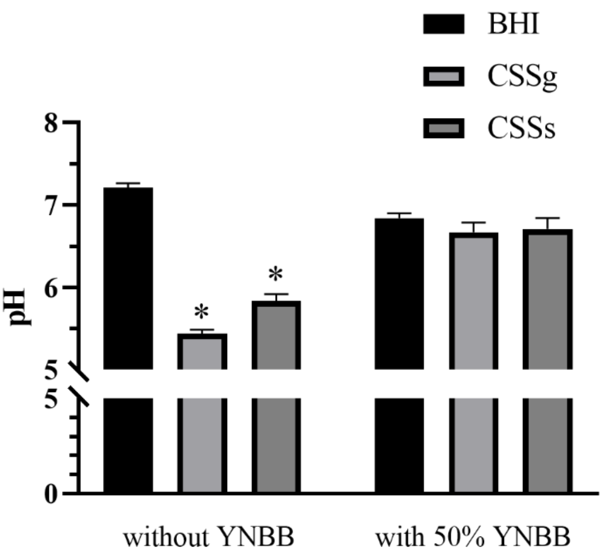

Figure S3. The pH values of CSSg and CSSs with or without 50% YNBB. BHI liquid medium was set as control. The values are shown as means ± SD from three independent experiments. * $P < 0.05$.

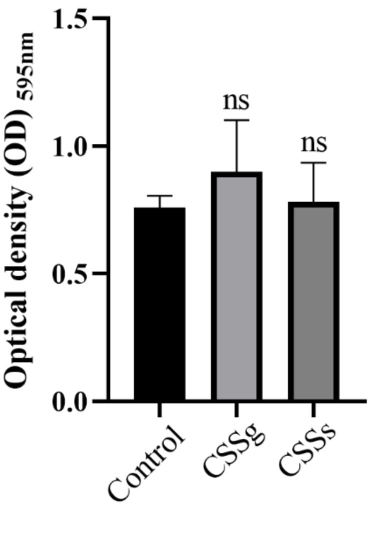

Figure S4. The effect of the culture supernatant of *S. gordonii* (CSSg) and *S. salivarius* (CSSs) on *C. albicans* biofilm formation. The biofilm biomass was investigated by crystal violet staining. ns: no significance. Control: 50% YNBB + 50% BHI. CSSg: 50% YNBB + 50% CSSg. CSSs: YNBB+50% CSSs.
